# Lipid specificity of the immune effector perforin

**DOI:** 10.1039/d0fd00043d

**Published:** 2020-08-13

**Authors:** Adrian W. Hodel, Jesse A. Rudd-Schmidt, Joseph A. Trapani, Ilia Voskoboinik, Bart W. Hoogenboom

**Affiliations:** Killer Cell Biology Laboratory, Peter MacCallum Cancer Centre 305 Grattan Street Melbourne VIC 3000 Australia ilia.voskoboinik@petermac.org; London Centre for Nanotechnology, University College London 19 Gordon Street London WC1H 0AH UK b.hoogenboom@ucl.ac.uk; Institute of Structural and Molecular Biology, University College London Gower Street London WC1E 6BT UK; Sir Peter MacCallum Department of Oncology, University of Melbourne Melbourne VIC 3000 Australia; Cancer Cell Death Laboratory, Peter MacCallum Cancer Centre 305 Grattan Street Melbourne VIC 3000 Australia; Department of Physics and Astronomy, University College London Gower Street London WC1E 6BT UK

## Abstract

Perforin is a pore forming protein used by cytotoxic T lymphocytes to remove cancerous or virus-infected cells during the immune response. During the response, the lymphocyte membrane becomes refractory to perforin function by accumulating densely ordered lipid rafts and externalizing negatively charged lipid species. The dense membrane packing lowers the capacity of perforin to bind, and the negatively charged lipids scavenge any residual protein before pore formation. Using atomic force microscopy on model membrane systems, we here provide insight into the molecular basis of perforin lipid specificity.

## Abstract 2

Physical membrane properties play a determining role in defining the sensitivity of membranes to the immune effector perforin.

## Graphical abstract

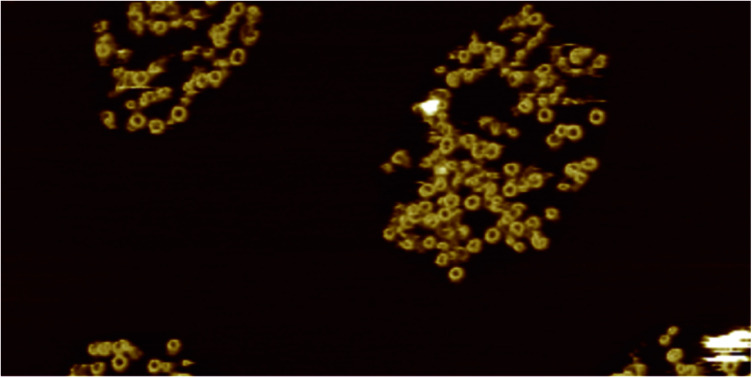

## Introduction

**Fig. 1:**
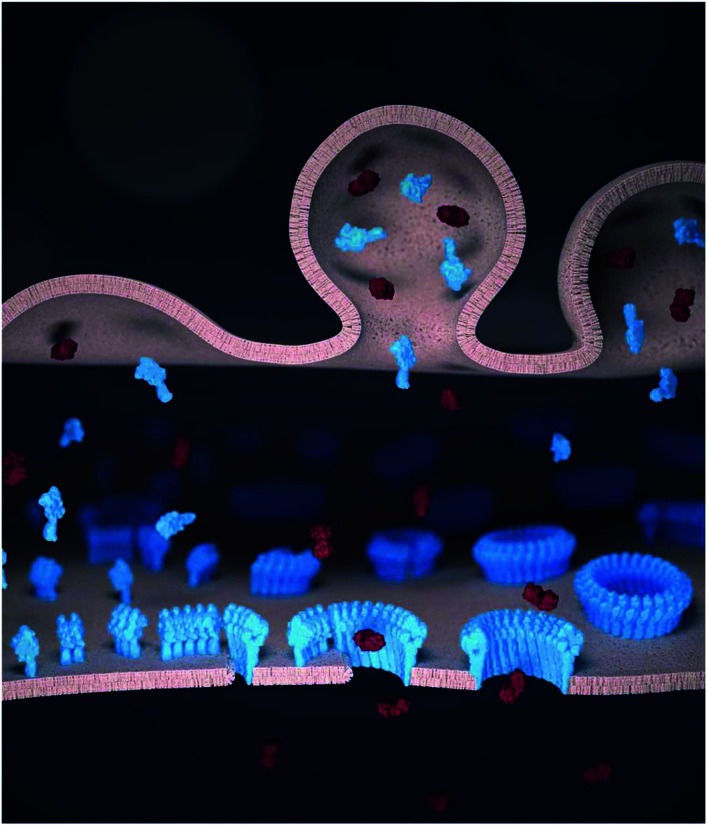
Schematic illustration of perforin pore formation and granzyme delivery in the synapse. Perforin (blue) and granzymes (red) are transported to the pre-synaptic membrane (top) by cytotoxic granules and released into the synaptic cleft. The (monomeric) perforin subsequently binds to the target cell membrane (bottom). On the target membrane, from left to right, perforin first oligomerizes into short, non-lytic perforin prepores. These prepores can convert to the pore state by inserting into the membrane, and subsequently recruit further prepores to sequentially grow the pore size. Once the pore size is sufficiently large, granzymes can diffuse into the target cells to trigger apoptosis.

Killer T cells or cytotoxic T lymphocytes (CTLs) kill virus-infected and cancerous cells to maintain immune homeostasis. During the immune response, CTLs form a synapse with their target cells in which they secrete the pore forming protein perforin and pro-apoptotic granzymes.^1,2^ Although both the CTL and the target cell plasma membrane are locally exposed at the synapse to perforin, perforin forms oligomeric pores in the target cell membrane but not in the CTL.^3^ Through the pores, granzymes can enter and trigger apoptosis in the target cell (Fig. 1). By contrast, the CTLs remain impermeable to granzymes and thus remain viable, and can sequentially kill multiple target cells.^3,4^ Without such resistance, CTLs (as well as natural killer cells) would be as vulnerable to perforin as the target cells. This would imply a (most costly) one-to-one ratio of killer cells to target cells and also prevent antigen experienced CTLs from differentiating into memory cells.

Perforin membrane binding – the first step in pore formation – is calcium-dependent and is mediated by its C2 domain.^5–9^ It was initially thought that phosphocholine lipids were perforin receptors in the target membrane,^10^ but it was later shown – by a comparison of lipids well above and just below their gel transition temperature – that lipid order was a more important factor in determining membrane sensitivity to perforin.^11^ The relatively tight plasma membrane packing of CTLs thus served as an explanation of the resistance of CTLs to perforin lysis. In the context of unidirectional killing in the immune synapse however, this hypothesis failed to explain the capability of CTLs to target and kill other CTLs, nor did it explain the absence of a clear correlation between the membrane packing of target cells and their susceptibility to perforin lysis.^12^ Following up on these early studies and on observations of perforin on model membranes by atomic force microscopy (AFM),^13^ we have recently revealed a two-layered lipid-based mechanism that renders CTLs refractory to perforin pore formation:^14^ firstly, increased lipid order and packing in the CTL membrane reduces perforin binding to the membrane, and secondly, perforin is sequestered and irreversibly inactivated by binding to the negative charge of externalised phosphatidylserine (PS) at the CTL surface. Importantly, these membrane changes are enhanced in the area of the CTL plasma membrane that is associated with the immune synapse.

This lipid specificity can be regarded in the context of other pore forming proteins in general^15^ and of the membrane attack complex-perforin/cholesterol dependent cytolysin (MACPF/CDC) family of pore forming proteins, which perforin is part of.^16–19^ The bacterial CDCs use – as implied by their name – cholesterol as a receptor on the membrane and only form pores in membranes above a rather sharp threshold of cholesterol contents, typically above ∼25–35%.^20–23^ This cholesterol dependence defines the specificity of CDCs to eukaryotic target cells, as bacteria generally do not contain cholesterol in their membranes.^24,25^ The mushroom derived MACPF pleurotolysin B utilizes the partner proteins ostreolysin A or pleurotolysin A to specifically bind and form pores in membranes containing sphingomyelin and cholesterol^26–28^ or insect specific lipids.^29^ In vertebrates, the membrane attack complex (MAC) is an immune effector that kills pathogenic bacteria. The formation of the MAC is facilitated on membranes that contain negatively charged lipids and show increased membrane tension, mimicking the surface of Gram-negative bacteria.^30^ Other examples from the vertebrate immune system are the more recently discovered pore forming proteins of the gasdermin family, which share some structural elements with MACPF/CDCs and trigger cell death by perforating the membranes of infected cells from the inside out.^31^ Gasdermin pore formation is related to negatively charged lipids that are in the inner leaflets of eukaryotic plasma membranes and mitochondria.

Besides cell-based assays, the lipid specificity of pore formation can be most conveniently studied on model lipid bilayers since these can be prepared from a wide selection of lipid components and therewith offer the ability to selectively alter biophysical properties. As a reference lipid, we used the dioleoyl derivative of phosphatidylcholine (PC), DOPC, which has a low liquid–gel transition, or melting, temperature (*T*_m_, *ca.* −17 °C (ref. 32)), and is therefore present in a liquid disordered (L_d_) state at physiological temperatures (37 °C). PC lipids in the L_d_ state (sometimes supplemented with cholesterol) are the most common components of model membranes used to visualize perforin assemblies,^7,13,14,33–35^ and the addition of cholesterol to L_d_ membranes increases membrane order and, at sufficiently high concentrations, it can give rise to a liquid ordered (L_o_) state.^25^ Below their melting temperature *T*_m_, lipids exist in a solid ordered (S_o_) gel state. Different lipids have different *T*_m_, *e.g.*, the dipalmitoyl derivative of PC, DPPC, has a *T*_m_ of *ca.* 41 °C (ref. 36) and is thus in the S_o_ state at physiological temperature. Over time, a membrane containing a mix of lipids can phase separate and display domains of different states of membrane order. Commonly used mixtures to mimic eukaryotic membranes use a low *T*_m_ lipid species like DOPC, cholesterol and high *T*_m_ sphingomyelin (SM), *e.g.*, egg SM. In such mixtures, one readily observes phase separation into PC-rich L_d_ domains and SM/cholesterol-rich L_o_ domains.^37,38^ Similarly, mixtures of DOPC and excess DPPC can lead to L_d_/S_o_ phase separation.^39^ The lipid phase state is an important factor to consider when mixing different types of lipids. Thus, to retain the L_d_ state of a reference DOPC bilayer, we can use the dioleoyl derivatives of phospholipids, *e.g.* dioleoyl phosphatidylserine (DOPS, *T*_m_*ca.* −11 °C (ref. 40)), ethanolamine (DOPE, *T*_m_*ca.* −8 °C (ref. 41)), or glycerol (DOPG, *T*_m_*ca.* −22 °C (ref. 42)).

Such model membranes can be prepared as supported lipid bilayers on a flat substrate, *e.g.*, mica or silica, facilitating their characterisation by in-liquid atomic force microscopy (AFM) experiments. AFM has become a popular tool to study the mechanisms of pore forming proteins,^43,44^ in part because it allows a relatively straightforward distinction between prepore assemblies and membrane inserted pores. This can be achieved either by detecting a height change^45,46^ or by the loss of mobility once the protein contacts the underlying substrate.^13,46^ Another important feature of AFM is its ability to distinguish between different lipid domains *via* Ångström-sized differences in membrane thickness,^47^ which allows us to simultaneously detect lipid phase boundaries and protein pores.

Here we use AFM-based experiments to expand upon our recent work^14^ on establishing and elucidating how perforin function depends on the physicochemical properties of the target membranes. Noting that perforin binding – and thus pore formation – is reduced on tightly packed membranes, we study and compare the effects of several properties that modulate membrane packing. In contrast to its response to changes in membrane order/packing, the interaction of perforin with the negatively charged DOPS is fundamentally different. The initial binding of perforin appears to be unaffected, but pore formation is disrupted. On model membranes, this effect is proportional to the amount of DOPS they contain. We therefore investigate how perforin interacts with pure DOPS membranes in the pursuit of understanding how perforin is deactivated by this lipid. Lastly, we describe the interaction of perforin with DOPE, as PE lipids are another major constituent of the plasma membrane.

## Experimental

### Recombinant proteins

Wild-type perforin (WT-PRF),^48^ disulphide locked perforin (TMH1-PRF),^13^ GFP fusion disulphide locked perforin (TMH1-GFP-PRF),^14^ and C2 domain mutant perforin (D429A-PRF)^5^ were expressed in baculovirus-infected Sf21 cells and purified from the supernatant as per the respective references provided. The CDC perfringolysin O (PFO) was kindly provided by Rana Lonnen and Peter Andrew (University of Leicester).

### Preparation of lipid vesicles and AFM samples

1,2-Dioleoyl-*sn-glycero*-3-phosphocholine (DOPC), 1,2-dipalmitoyl-*sn-glycero*-3-phosphocholine (DPPC), 1,2-dioleoyl-*sn-glycero*-3-phosphoethanolamine (DOPE), 1,2-dioleoyl-*sn-glycero*-3-phospho-(1′-rac-glycerol) (DOPG), 1,2-dioleoyl-*sn-glycero*-3-phospho-l-serine (DOPS), egg sphingomyelin (egg SM) and cholesterol were purchased as powders from Avanti Polar Lipids (Alabaster, AL, USA). Where indicated, the lipids were mixed in the desired molar ratios (±5% confidence intervals). Note that the provided mixing ratios do not necessarily represent the lipid concentration displayed on the final bilayer surface. The effective exposure of negatively charged lipids on the surface of supported lipid bilayers can be lower by a factor of 2 or more due to interactions with the substrate.^49^ At a concentration of 0.5–1 mg mL^−1^, unilamellar vesicles with a nominal diameter of 100 nm were prepared using the lipid extrusion method.^13,50^

4–8 μL of the unilamellar vesicles (containing 4 μg of lipid) were added to a freshly cleaved, ∅ 10 mm mica disc (Agar Scientific, Stansted, UK) and topped up with 80 μL of adsorption buffer containing 150 mM NaCl, 25 mM Mg^2+^, 5 mM Ca^2+^, and 20 mM HEPES, pH 7.4. To form a pure DOPG bilayer, lower salt conditions were necessary^51^ and the buffer was thus adjusted, instead containing no Mg^2+^ and 10 mM Ca^2+^. The lipids were incubated for 30 minutes above the *T*_m_ of the constituent lipids to cover the mica substrate with an extended lipid bilayer. Excess vesicles were removed by washing the bilayer 6–12 times with 80 μL of the adsorption buffer.

Additional washes were applied to samples that contained DOPS, for which we found that Mg^2+^ in the buffer interfered with perforin binding, samples that contained DOPG to remove excess Ca^2+^, or to control samples that required the removal of Mg^2+^: these were washed an additional 6 times with 80 μL buffer containing 150 mM NaCl, 5 mM Ca^2+^, and 20 mM HEPES, pH 7.4.

Wild-type perforin (WT-PRF), disulphide-locked perforin (TMH1-PRF), C2 domain mutant perforin (D429A-PRF) and perfringolysin O (PFO) were diluted up to *ca.* ten-fold to a volume of 40 μL in 150 mM NaCl and 20 mM HEPES, pH 7.4, and injected onto the sample, to reach concentrations of 150 nM or, where noted, *ca.* 400 nM above the model membrane. The protein was incubated for 2 (where noted) or 5 min at 37 °C. To unlock TMH1-PRF after its binding to the membrane, the engineered disulphide bond was reduced by addition of 2 mM DTT (Sigma-Aldrich, St. Louis, MO, USA) and 10 min incubation at 37 °C. We previously verified that, once TMH1-PRF was bound to target membranes, the effect of DTT on its native disulphide bonds did not change the pore forming functionality.^13^ Mobile TMH1-PRF assemblies were fixed by addition of glutaraldehyde 8% EM grade (TAAB Laboratories, Aldermaston, UK) to a final concentration of 0.04% v/v, and 10 min incubation at room temperature. Fixed TMH1-PRF assemblies were removed from their DOPS membrane substrate by chelating calcium with EGTA. To this end, the samples were washed 6 times with 80 μL of buffer containing 150 mM NaCl, 20 mM HEPES, and 4 mM EGTA, pH 7.4. EGTA was immediately removed from the sample afterwards by 6 further washes with 80 μL buffer containing 150 mM NaCl, 5 mM Ca^2+^, and 20 mM HEPES, pH 7.4.

### AFM imaging and analysis

AFM images were recorded by either force–distance curve-based imaging (PeakForce Tapping) on a MultiMode 8 system (Bruker, Santa Barbara, CA, USA) or photothermal excitation (blueDrive) on a Cypher ES AFM (Oxford Instruments, Abingdon, UK). The imaging conditions with commercial MSNL cantilevers (Bruker) for PeakForce Tapping are outlined in ref. 14. In brief, PeakForce Tapping was performed at 2 kHz and a maximum tip–sample separation of 5–20 nm. Images were recorded at 0.75 Hz scan speed and tip–sample interaction forces between 50 and 100 pN on an E-Scanner (Bruker, Santa Barbara, CA, USA) with temperature control. For blueDrive, we used BL-AC40TS probes (Olympus, Tokyo, Japan). The UV laser for photothermal excitation was focussed onto the cantilever base. The laser was tuned to the resonance frequency of the cantilever in liquid (*ca.* 25 kHz) and the amplitude was adjusted to 1 V. Imaging was performed at an amplitude setpoint of *ca.* 750 mV and 1 Hz scan speed. All samples were imaged at 37 °C to retain thermotropic properties, or at room temperature.

**Fig. 2:**
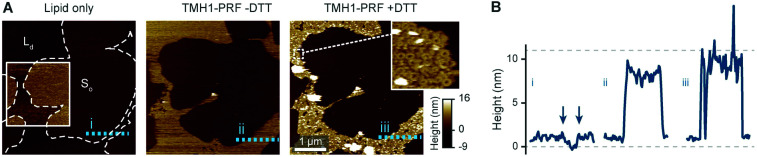
Prepore-locked TMH1-PRF preferentially binding to L_d_ domains in a phase separated L_d_/S_o_ membrane. (A) AFM images of a supported lipid bilayer composed of DOPC/DPPC mixed in a 1 : 7 molar ratio. The first panel (‘Lipid only’) shows the empty membrane, where the colour contrast has been enhanced in the inset (colour scale: 4 nm) to better visualize the lipid phase separation. The phase boundaries are outlined for the whole image by dashed white lines. Addition of TMH1-PRF (‘TMH1-PRF − DTT’) leads to the formation of a diffuse plateau limited to L_d_ domains. Similar plateaus were earlier interpreted as mobile prepore assemblies.^13^ After addition of DTT (‘TMH1-PRF + DTT’), the mobile assemblies insert into the membrane, and a dense layer of arc- and ring-shaped pores is formed (see inset), still confined to L_d_ domains. Size of the inset, 150 nm. (B) Height profiles extracted along the dashed coloured lines in (A). The profiles depict the 0.5–1 nm height change at the phase boundaries (i) (the boundaries are highlighted by arrows), and the *ca.* 7–11 nm tall prepore and pore layers (ii and iii). Dashed grey lines indicate the height of the L_d_ membrane (0 nm) and the height of a perforin monomer (11 nm (ref. 7)). Note that perforin features can appear compressed due to tip–sample interaction forces. The data was recorded at 37 °C.

Raw AFM images were background subtracted with reference to the lipid surface, masking perforin and applying second-order flattening. Height values of perforin prepores/pores indicated in the manuscript are given with ±1 nm confidence intervals, with the uncertainty due to scanner calibration and possible sample deformation caused by the probe–sample interaction. The same colour/height scale was applied to all images (except for the insets in Fig. 2A and 4A as specified in their captions), spanning 25 nm and 9 nm below and 16 nm above the membrane surface (set to 0 nm). The colour scale is only depicted once, in Fig. 2. Values for perforin coverage were estimated either by the area above a height threshold located 6–8 nm above the membrane surface and adjusted to counteract tip broadening effects; or, when sufficient images at a higher pixel resolution were available, by tracing pore shapes with 3dmod 4.9.4 (BL3DEMC & Regents of the University of Colorado^52^). Perforin coverages obtained by both methods are normalized with respect to a 100% DOPC reference and given as values between 0 and 1. One-way analysis of variance (ANOVA) with Dunnett’s post-hoc analysis was performed in R-3.6.3 using the multcomp package.^53^

### Perforin binding to lipid strips

Membrane lipid strips (Echelon Biosciences, Salt Lake City, UT, USA) were incubated in 4 mL of blocking buffer containing 3% w/w BSA (Roche Diagnostics GmbH, Mannheim, Germany), 150 mM NaCl, and 20 mM HEPES, pH 7.4 for 1 h at room temperature. 2 μg mL^−1^ TMH1-GFP-PRF was added to a lipid strip in 4 mL of blocking buffer supplemented with 2 mM CaCl_2_, pH 7.4. The use of the GFP fusion construct allowed readout of the lipid strips without the need for antibody labelling. To assess calcium-independent (non-specific) perforin binding, 2 μg mL^−1^ TMH1-GFP-PRF in 4 mL of blocking buffer was added to a lipid strip. After 1 h of incubation at room temperature, the lipid strips were washed three times with 4 mL of blocking buffer (with or without adding 2 mM CaCl_2_ to match the initial incubation). GFP fluorescence (of wet lipid strips) as a measure of perforin binding was recorded on an iBright 1500 western blot imaging system (Thermo Fisher Scientific, Waltham, MA, USA). The strips were stored in the blocking buffer for *ca.* 96 hours at 4 °C and imaged again.

## Results and discussion

### Effect of lipid order on perforin binding and pore formation

To visualize the binding of perforin to different phase domains of different levels of lipid order, we used a disulphide-locked mutant, TMH1-PRF, that can bind to and assemble on, but not insert into the target membrane.^13^ Its pore forming functionality was fully restored after reducing the disulphide bond with the reducing agent dithiothreitol (DTT). This mutant has an advantage over the wild-type protein in that membrane binding and pore insertion can be uncoupled and studied as two separate events.^13,14^

Using TMH1-PRF, we first verified the earlier observation^11^ that perforin does not bind to lipids that are below their gel-transition temperature, *i.e.*, in the gel or S_o_ phase. This could be best articulated by visualizing the binding of perforin on phase separated bilayers that contained both L_d_ and S_o_ domains. To this end, we mixed high *T*_m_ DPPC and low *T*_m_ DOPC and verified the phase separation by AFM, with the S_o_ domains appearing *ca.* 1 nm higher than the L_d_ domains (see Fig. 2). Upon exposure to TMH1-PRF, the L_d_ domains showed extensive protein coverage by the appearance of diffuse plateaus with a height close to 10 nm above the membrane. As in previous work, we interpret these plateaus as membrane-bound but not inserted and, hence, highly mobile perforin prepores.^13^ After addition of DTT, the TMH1-PRF transitioned from the prepore to the membrane-inserted pore state, while remaining localized within L_d_ domains.

**Fig. 3:**
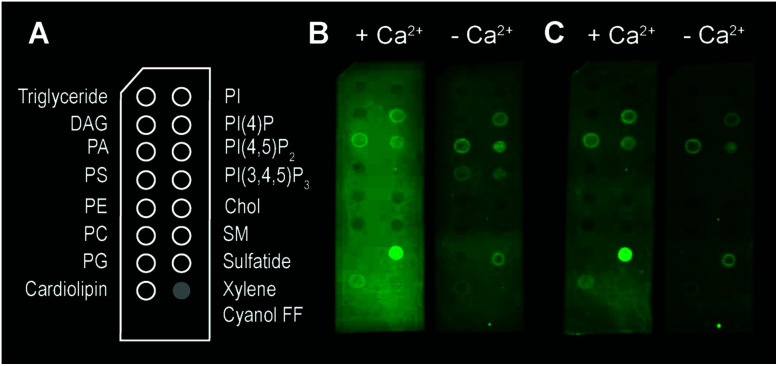
Binding of TMH1-GFP-PRF to lipid strips in the presence and absence of 2 mM Ca^2+^, visualized by fluorescence imaging.† (A) Schematic layout of the lipid strips, with spots of lipids blotted where indicated (xylene cyanol FF is a non-lipid control). (B) Detection of TMH1-GFP-PRF binding to lipid strips in the presence and absence of Ca^2+^ (‘+Ca^2+^’ and ‘−Ca^2+^’ respectively) immediately after washing. (C) The same lipid strips as in (B) after 4 days at 4 °C in blocking buffer.

Besides bilayer-based experimental substrates, membrane strips blotted with different types of lipids have been used to characterize the lipid specificity of several pore forming proteins (*e.g.*ref. 29 and 54–56). We find that commercially available strips fail to detect perforin binding to, for example, PC (Fig. 3), in agreement with previous reports using lipid strips;††Some of the observed perforin–lipid binding was different from previously published results. Without investigating this further, we point out that we used a recombinant mouse perforin mutant (*vs.* native human perforin elsewhere) and detected fluorescence directly (*vs.* primary/secondary antibody detection elsewhere). Furthermore, we noted some perforin binding that exclusively occurred in the absence of Ca^2+^ and disappeared after prolonged incubation in the washing buffer. The reasons for this are unknown to us.^55^ this is in apparent contradiction to the scientific literature spanning from the 1980s^10^ to today.^14^ This contradiction can be simply explained by noting that the blotted (phospho-) lipids have a high *T*_m_ and were not in an L_d_ state at the physiological/experimental temperature, and are thus likely to cause erroneous readings when lipid order is a factor of importance for protein binding (such as for perforin). Moreover, lipids that bound perforin on these lipid strips (Fig. 3B and C, ‘+Ca^2+^’ *vs.* ‘−Ca^2+^’) did so independently of the calcium that is required for perforin binding to target membranes; such interactions may therefore be artefacts due to defects in the lipid covered membrane surfaces.

**Fig. 4:**
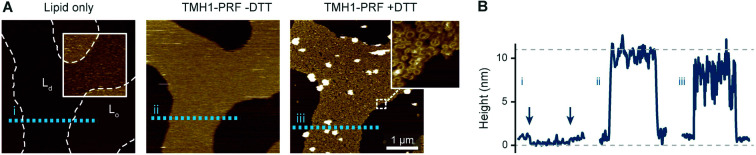
TMH1-PRF preferentially binding to L_d_ domains in a phase separated L_d_/L_o_ membrane, analogous to Fig. 2. (A) AFM images of an approximately equimolar DOPC/egg SM/cholesterol supported lipid bilayer. ‘Lipid only’ shows the empty membrane with the phase boundaries between L_d_ and L_o_ domains highlighted by the inset (with a 4 nm colour scale) and dashed white lines. TMH1-PRF exclusively binds L_d_ domains (‘TMH1-PRF − DTT’) and remains (mostly) confined there after addition of DTT (‘TMH1-PRF + DTT’) and the formation of transmembrane pores (see inset). Size of the inset, 150 nm. (B) Height profiles extracted along the dashed coloured lines in A. The profiles depict the 0.5–1 nm height change at the phase boundaries (i) (the boundaries are highlighted by arrows), and the *ca.* 7–11 nm tall prepore and pore layers (ii and iii). Dashed grey lines indicate the height of the L_d_ membrane (0 nm) and the height of a perforin monomer (11 nm (ref. 7)). Note that perforin features can appear compressed due to tip–sample interaction forces. The data was recorded at 37 °C. Figure reproduced from ref. 14, under a Creative Commons Attribution 4.0 International License (CC BY 4.0).

As reported previously^14^ and reiterated here for completion and for comparison with Fig. 2, we observe the same preference of perforin for L_d_ domains when adding TMH1-PRF to phase separated lipid membranes that contain both L_d_ and L_o_ domains,^14^ which is also in agreement with previous results using WT-PRF.^13,14^ As shown in Fig. 4, perforin again preferentially binds to and forms pores in L_d_ domains, albeit that some rare examples of perforin binding may be observed on L_o_ domains. This binding dependence on lipid order is also in agreement with the reduction of WT-PRF pore formation on highly ordered egg sphingomyelin membranes compared with L_d_ 18 : 1 sphingomyelin membranes.^14^

In the experiments reported above, lipid order was varied by using lipids with identical headgroups but different hydrophobic tails. In addition, membrane order can be dependent on divalent ions that intercalate with lipid headgroups, modulating intermolecular attractions.^57^ In most of our AFM work on model membranes, we used up to 25 mM Mg^2+^ in our buffers to stabilize the supported lipid bilayers on the negatively charged mica substrate. This concentration is about one order of magnitude higher than blood levels.^58^ To test how the presence of Mg^2+^ affects perforin function, we designed experiments in which we washed samples to remove Mg^2+^ from the buffer before adding perforin (WT-PRF, at 37 °C as usual) onto model membranes in either the L_d_ (pure DOPC), L_o_ (DOPC/cholesterol or egg SM/cholesterol, both 47/53 molar ratio), or S_o_ (pure egg SM) state.^37^

**Fig. 5:**
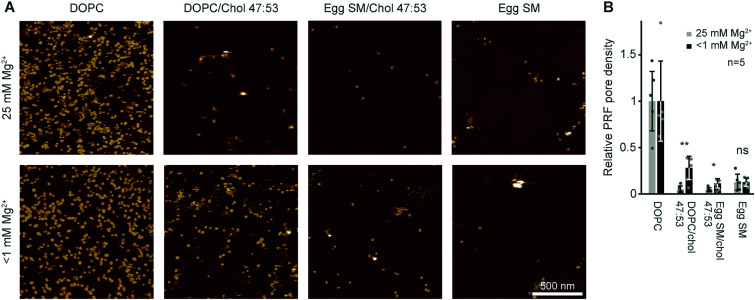
Perforin lipid specificity as a function of Mg^2+^ concentration. (A) Representative AFM images of WT-PRF pores incubated on magnesium-depleted (<1 mM Mg^2+^, see Experimental) membranes of different lipid compositions and at 25 mM Mg^2+^, as indicated. The data was recorded at 37 °C. (B) Average perforin pore formation on different lipid mixtures, at Mg^2+^ concentrations of 25 mM and <1 mM, normalized to the number of pores on DOPC-only membranes. Here, perforin was incubated for 2 min instead of 5 min (see Experimental) to match the experimental conditions of the two datasets. Error bars represent standard deviations. The statistical significance was assessed using ANOVA with Dunnett’s post-hoc analysis, where ‘ns’ is not significant, *p < 0.05, **p < 0.01. The data for 25 mM Mg^2+^ are reproduced from ref. 14.

By comparison with previously published data acquired in the presence of Mg^2+^, we found the differences between pore formation at high and low levels of Mg^2+^ to be mostly insignificant (see Fig. 5). However, for low levels of Mg^2+^, a significant but small increase in pore formation was found on the L_o_ membranes consisting of DOPC/cholesterol and egg SM/cholesterol. We did not note any phase separation in any of these lipid substrates at either level of Mg^2+^, suggesting that perforin binding was uniformly affected, if at all, in all samples. In summary, the suggested increase in membrane order due to Mg^2+^ may be present and affect perforin pore formation in membranes of intermediate order, but this effect is small compared to the effects on lipid order due to high amounts of cholesterol or introduction of gel-phase lipids as reported above.

### Effect of lipid charge on perforin pore formation

By the here described variations in perforin binding with lipid order, we can explain the reduced binding of perforin to CTLs that has been shown in earlier work.^14^ However, when incubated with higher concentrations of recombinant perforin, CTLs were found to still resist perforin pore formation in spite of binding amounts of perforin that were lytic to target cells, which we attributed to the presence of PS in the outer leaflets of the lymphocyte membranes.^14^

**Fig. 6:**
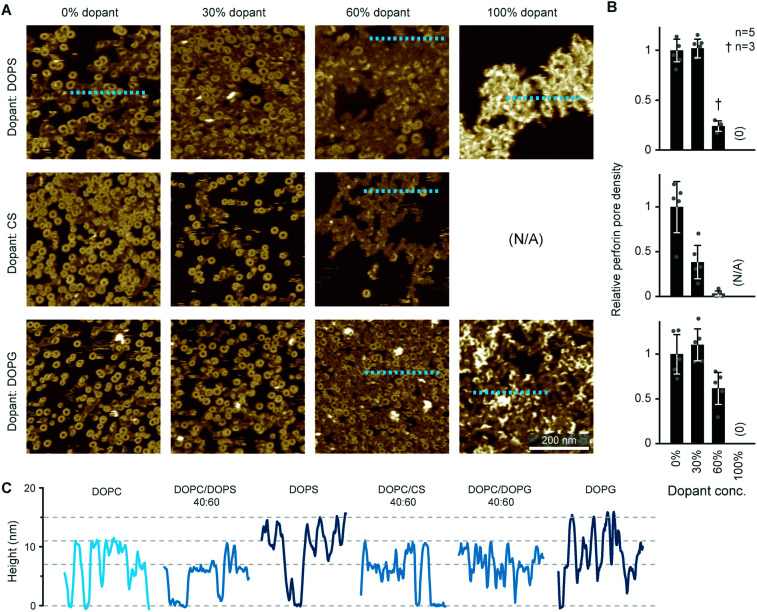
WT-PRF pore formation on different substrates containing DOPC and varying levels of either DOPS, DOPG, or cholesterol sulphate (CS). (A) Representative AFM images of perforin pores and aggregations on the different substrates. For pure CS, no bilayer could be formed. All data was recorded at room temperature. (B) Quantification of pore formation (mean ± SD) in the samples shown in (A), relative to the 0% dopant/100% DOPC reference. (C) Height profiles extracted along the dashed lines in (A); the ‘DOPC’ reference profile was extracted from the first tile in (A). The different profiles show the membrane level adjusted to 0 nm and the height of perforin pores (*ca.* 11 nm) and aggregates (*ca.* 7 nm at 60% dopant levels, up to 15 nm at 100% dopant levels), as highlighted by horizontal dashed lines. The colour tone of the profiles is darker compared to the ‘DOPC’ reference, corresponding to the level of negative charge present in the membrane substrates. Panels (A) and (B) are reproduced from ref. 14, under a Creative Commons Attribution 4.0 International License (CC BY 4.0).

Perforin can bind to PS-rich membranes, but pore formation is decreased: instead of pores, perforin aggregates into dysfunctional plaques.^14^ PS lipids have a net negative charge at physiological pH, and we previously hypothesized that this negative charge is the underlying cause of perforin dysfunction. We therefore tested the effect of the negatively charged DOPG and cholesterol sulphate on perforin pore formation. As predicted, the decrease in perforin pore formation was proportional to the levels of negatively charged lipids in the membranes and, possibly, further affected by an ordering effect induced by cholesterol sulphate (Fig. 6 A and B). This leads us to conclude that it is a generic negative surface potential, rather than specific lipid headgroups, that prevent perforin pore formation here.

On the membranes with higher negative charge, the decrease in WT-PRF pore formation was accompanied by an increase in the presence of plaques of protein aggregates. Perhaps the most striking feature of these plaques is their height. Perforin aggregations appear at *ca.* 7 nm in height when PC is doped with 30–60 mol% negatively charged lipids; of note, the actual proportion of negatively charged lipids in the outer layer of supported lipid bilayers is likely to be reduced by at least one half due to leaflet asymmetry in negatively charged supported lipid bilayers.^49^ However, on pure DOPS and DOPG membranes, the aggregations appear as plaques with a height of (up to) 15 nm above the membrane surface (Fig. 6C). This height is to be compared with the *ca.* 11 nm height of membrane-bound perforin prior to and after membrane insertion.^13,14^

**Fig. 7:**
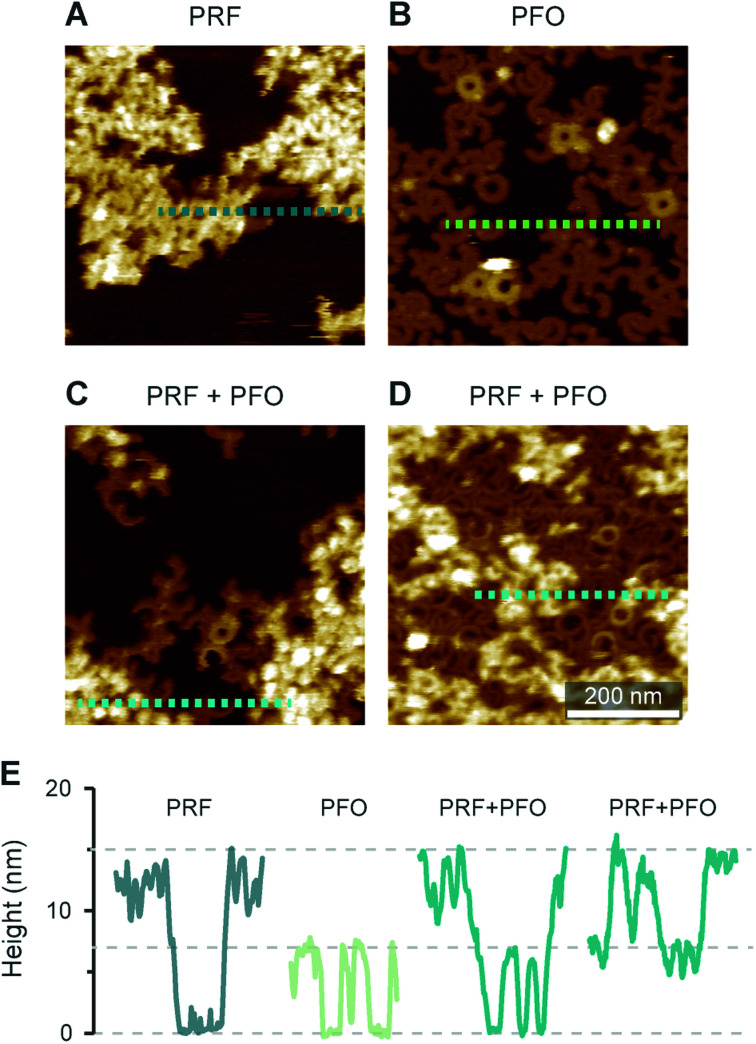
AFM images of WT-PRF and PFO on 70 : 30 mol% DOPS/cholesterol bilayers for height referencing. (A) WT-PRF forms protein plaques on the lipid bilayer. (B) Cholesterol dependent PFO pore formation, visible as arc- and ring-shaped assemblies. A small number of the assemblies appeared higher, probably due to incomplete membrane insertion: PFO collapses from *ca.* 10 nm to 7 nm height upon membrane insertion.^45^. (C and D) When the DOPS/cholesterol bilayers were incubated first with WT-PRF and next with PFO, perforin plaques were observed adjacent to PFO pores. We here show two samples incubated with different amounts of PFO, *ca.* 150 nM in (C) and *ca.* 450 nM in (D). Consequently, the membrane surface is still visible in (C), while in (D) the PFO pores cover most of the remaining membrane. (E) Height profiles extracted along the dashed lines in (A)–(D). Horizontal lines at 0, 7, and 15 nm highlight the membrane surface and the heights of PFO pores and perforin plaques, respectively. All AFM data were recorded at room temperature.

Intriguingly, the observed 15 nm height above the membrane agrees with the full height of perforin pores including the hairpins that span the membrane.^7^ This suggests that the protein has initiated the transition from its prepore to pore state, yet while unfurling these hairpins, it has failed to insert into the membrane. Given this possible interpretation, we sought to first further verify the height measurements of perforin plaques on pure PS membranes, by including the cholesterol dependent cytolysin (CDC) perfringolysin O (PFO) as a height ruler in our experiments. Like other CDCs, PFO forms pores that protrude *ca.* 7 nm above cholesterol-containing membranes.^45,46,59,60^ To this end, we prepared 70 : 30 mol% DOPS/cholesterol membranes, on which perforin behaved similarly as on pure DOPS membranes (Fig. 7A) but which – by the inclusion of cholesterol – allowed CDC binding and pore formation too (Fig. 7B). By first incubating these membranes with perforin and next with the CDC PFO, we observed PFO pores in addition to perforin plaques (Fig. 7C and D), with the perforin plaques being approximately double the height above the membrane as the PFO pores, which were taken as a height reference of *ca.* 7 nm (Fig. 7E). This fully confirms the extraordinary height of perforin on DOPS-only bilayers.

**Fig. 8:**
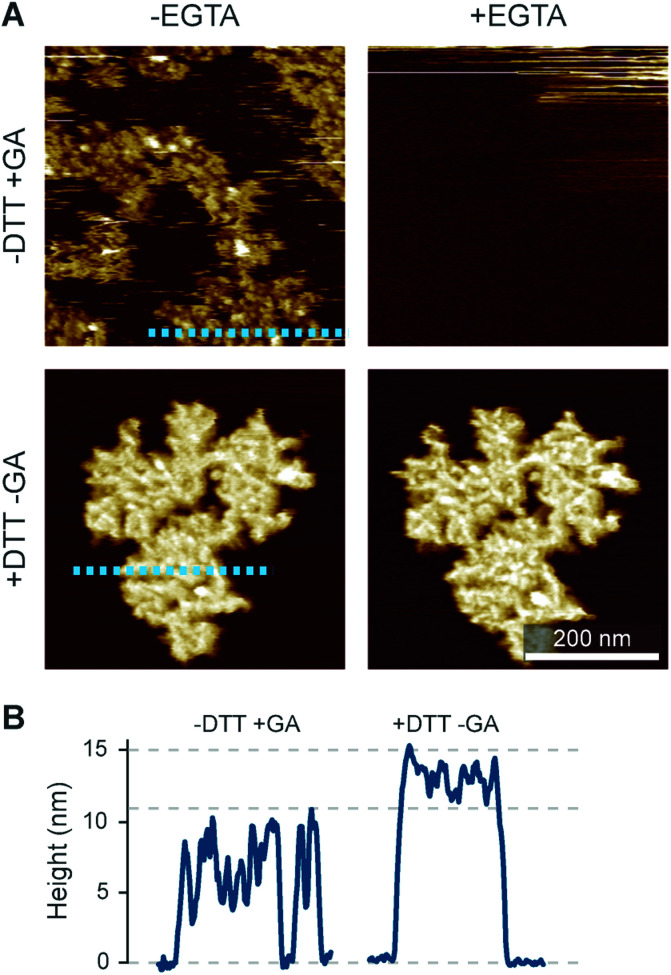
Membrane binding to DOPS membranes prior to plaque formation, assessed with TMH1-PRF. (A) AFM images of locked and unlocked TMH1-PRF on DOPS membranes. To visualize initially mobile TMH1-PRF features (not shown here) in the AFM images, we fixed the protein by addition of glutaraldehyde (GA). The patches of crosslinked perforin (‘−DTT + GA’, ‘−EGTA’) are removed by washing the samples with the calcium-chelating agent EGTA (‘−DTT + GA’, ‘+EGTA’). If TMH1-PRF is unlocked by addition of DTT, perforin plaques are formed similarly to WT-PRF (‘+DTT − GA’, ‘−EGTA’). The plaques are not visibly affected by washing with EGTA (‘+DTT − GA’, ‘+EGTA’). (B) Height profiles of cross-linked (‘−DTT + GA’) and unlocked (‘+DTT − GA’) TMH1-PRF extracted from the panels in A, as indicated by dashed lines. All images were recorded at room temperature. The panels in (A) are reproduced from ref. 14, under a Creative Commons Attribution 4.0 International License (CC BY 4.0).

The preconditions and structural changes necessary to form such perforin plaques are unknown. In our earlier studies with the non-functional TMH1-PRF on pure DOPS bilayers, it emerged that in the membrane-binding and early assembly stage, *i.e.*, before pore insertion, the behaviour of perforin is similar to that observed on DOPC bilayers:^13,14^ TMH1-PRF on DOPS and DOPC (i) showed a similar distribution of subunits per assembly (quantification in ref. 14), (ii) was *ca.* 10 nm in height above the membrane (similar to the height of an upstanding perforin molecule), and (iii) diffused freely and could be removed from the membrane surface by chelation of Ca^2+^ (demonstrated by the removal of oligomers after chelating calcium from the buffer, see Fig. 8A). After adding DTT and thus unlocking TMH1-PRF, the short oligomers clustered together and increased their height to *ca.* 15 nm (Fig. 8B). Taken together, this supports the interpretation that the formation of plaques on PS is linked to the unfurling of the protein as it attempts – unsuccessfully – to insert into the membrane.

**Fig. 9:**
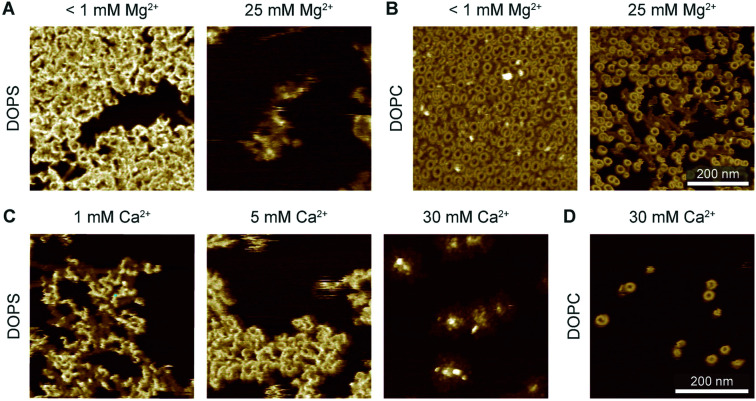
Interaction of WT-PRF with DOPS membranes at various levels of Ca^2+^ and Mg^2+^. (A) WT-PRF on DOPS membranes at low (<1 mM) and high (25 mM) Mg^2+^ levels in the buffer. Perforin only forms the *ca.* 15 nm high plaques at low Mg^2+^ levels, although some protein binds at a high Mg^2+^ level. Here, we used 400 nM WT-PRF (instead of the 150 nM used for other experiments, see Experimental) to test the effect at high perforin concentrations. (B) Analogous experiment to (A) on DOPC instead of DOPS membranes, as a control. At both low and high Mg^2+^ concentrations, arc- and ring-shaped perforin pores are visible.‡‡We note that in the images depicted here, the overall perforin coverage at high Mg^2+^ concentration appears lower (at 400 nM WT-PRF concentration). It is not clear if this difference is significant; to date, we do not have sufficient AFM data and repeats of this experiment to rigorously quantify the number of perforin pores at high *versus* low Mg^2+^ concentrations. (C) Perforin plaques formed on DOPS membranes at Ca^2+^ concentrations of 1, 5, and 30 mM Ca^2+^ (with no Mg^2+^). For larger Ca^2+^ concentrations, the plaques appear less dispersed. (D) For comparison, arc- and ring-shaped pores formed on DOPC at 30 mM Ca^2+^. All images were recorded at room temperature.

To further investigate how this behaviour depends on electrostatic interactions, we varied the concentration of divalent ions in solution, thus changing the screening of surface charges. Firstly, when 5 mM Ca^2+^ and an excess concentration of Mg^2+^ (25 mM) were present in the buffer, perforin (WT-PRF) would not bind to or form plaques on a DOPS membrane, even at higher perforin concentrations (Fig. 9A and B). Secondly, in the absence of Mg^2+^, the appearance of the plaques was dependent on the Ca^2+^ concentration in the buffer: at higher concentrations of Ca^2+^, there was a decrease in the spread of plaques over the membrane surface (Fig. 9C and D). The dependence of perforin membrane binding on the concentration of divalent cations further confirms that the observed behaviour on DOPS is mediated by electrostatic interactions. These two observations are different from what we observe on DOPC membranes, where a similar increase in Ca^2+^ concentration produced no effect on the formation of arc- and ring-shaped pores.

**Fig. 10:**
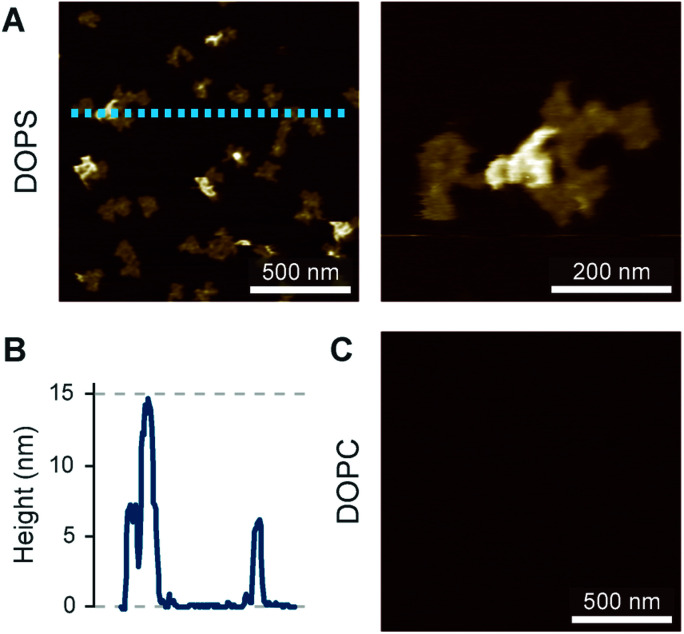
D429A-PRF, a perforin with mutated C2 domain, binds to DOPS but not DOPC membranes. (A) D429A-PRF forming plaques on a DOPS membrane. (B) Height profile extracted along the dashed line in (A). (C) A DOPC membrane incubated with the same amount of D429A-PRF as in (A) does not show perforin binding. AFM data was recorded at room temperature.

For functional perforin, the initial membrane binding occurs through its C2 domain. By mutating this domain in D429A-PRF,^5^ we could also test whether the initial perforin binding depends on lipid composition: the mutation completely abrogated D429-PRF binding to DOPC bilayers, but on DOPS membranes the mutant still formed plaques with heights of mostly *ca.* 7 nm and up to 15 nm (Fig. 10), roughly consistent with the plaques formed by WT-PRF (Fig. 6).

Taken together, the experimental data indicate that negatively charged membranes disrupt perforin function due to electrostatic interactions, and that the disruptions are manifested at the stage of membrane insertion. The data in Fig. 8 and 9 show that the interaction of perforin with PS membranes depends on calcium, suggesting the involvement of calcium binding sites within the C2 domain.^9^ As illustrated by locked TMH1-PRF in Fig. 8, perforin initially binds to PS membranes in an upright orientation, further indicating that the C2 domain is facing the membrane surface (an alternative, but somewhat far-fetched hypothesis is that the protein is bound upside down). In contrast, the C2 domain mutant perforin shows different binding to PS membranes compared to PC membranes, *i.e.*, binding is completely absent on PC membranes. To reconcile the seemingly contradictory data, we can imagine two scenarios: (I) disruption of perforin function is caused by small differences in the binding geometry between the C2 domain and charged lipid substrates,§§D429A-PRF is distinct from WT in its dual effect on the C2 domain: it is unable to bind two out of five C2 domain Ca^2+^ ions,^9^ and it also fails to undergo the conformational change required for the reorientation of W427 and Y430 residues – a critical Ca^2+^-dependent step required for perforin binding to a membrane.^8^ The fact that D429A binds to PS, but not to PC, suggests that perforin binding to PS occurs through a non-canonical mechanism that is independent of the hydrophobic interactions of W427, Y430, Y486 and W488 with the membrane.^8^ leading to protein misfolding and plaque formation; (II) only a (possibly small and imperceptible in AFM images) fraction of perforin is required to bind DOPS in a different fashion, possibly independent of the C2 domain, and this fraction disables otherwise correctly bound perforin when it attempts to insert into the membrane. Of note, a similar disruptive effect was observed when functional WT-PRF was co-incubated with excess non-functional TMH1-PRF,^13^ although in that case, the pore forming functionality could be fully restored by subsequent addition of DTT (unlocking the disulphide lock in TMH1-PRF).

### Effect of membrane tension on perforin pore formation

Besides lipid order and charge,^14^ another physical membrane property that may modulate perforin pore formation is membrane tension, which has been suggested to enhance perforin function in the immune synapse.^61^ To some extent, such effects can be tested in supported lipid bilayers by the inclusion of curvature-inducing lipids. For example, phosphatidylethanolamine (PE) is a zwitterionic lipid with no net charge and a relatively small headgroup compared with the width of its hydrophobic tail. This causes PE to favour curved membrane arrangements, consistent with its prevalence in the inner leaflet of the eukaryotic plasma membrane and implying interfacial tension when forced to arrange in planar membranes; indeed, bilayers containing only (unsaturated) PE lipids do not form under physiological conditions.^62^ PE can be synthesized from PS by decarboxylation and is co-located with PS in the inner plasma membrane leaflet;^63,64^ their externalization is regulated by the same transporters.^65^

**Fig. 11:**
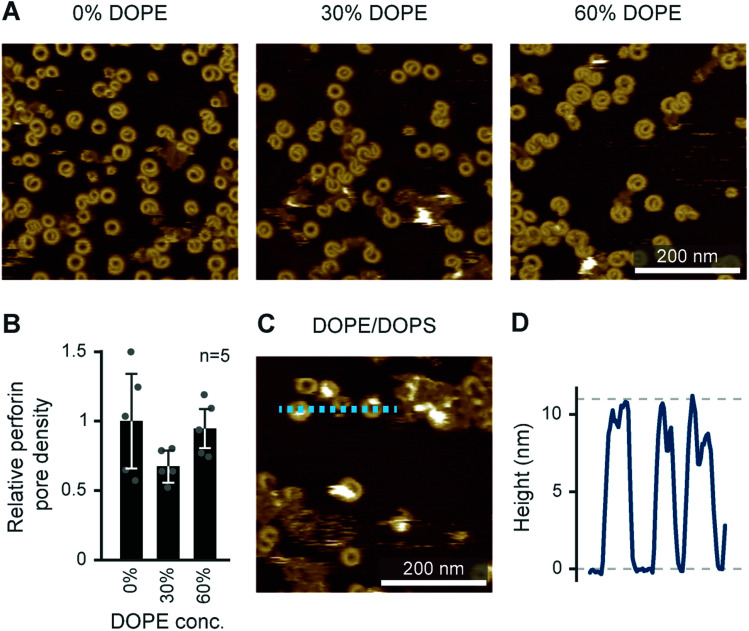
Effect of DOPE on WT-PRF pore formation. (A) AFM images of perforin pore formation on membranes containing DOPC and increasing amounts of DOPE. (B) Quantification of the pore formation normalized to the 0% DOPE/100% DOPC reference. Error bars represent standard deviations. (C) AFM image of perforin on an 80 : 20 DOPS/DOPE membrane, showing at least partial restoration of pore formation. (D) Height profile extracted along the dashed line in (C). AFM images were recorded at room temperature.

To test how the addition of PE affects pore formation by perforin, we doped a DOPC bilayer with up to 60 mol% DOPE, and exposed the resulting membranes to WT-PRF. As shown in Fig. 11A and B, the addition of DOPE had no significant influence on the pore forming capacity of perforin *per se*. We also performed an alternative experiment in which we tested whether addition of 80 mol% DOPE would restore pore forming capacity in a DOPS host bilayer. A qualitative assessment of the AFM images indeed indicated that the inclusion of PE caused some restoration of pore formation on DOPS membranes (Fig. 11C). This preliminary result could be explained by presuming that perforin directly binds to PE lipid headgroups. However, it is also possible that PE, with its small headgroup, provides no direct perforin binding site and, instead, introduces membrane defects that expose areas otherwise buried underneath the membrane surface or generally acts as a spacer between DOPS molecules. As a result, perforin might access and bind the PS headgroup differently, thus (partially) restoring its functionality. Future experiments will need to determine if perforin can bind PE directly.

## Conclusions

**Fig. 12:**
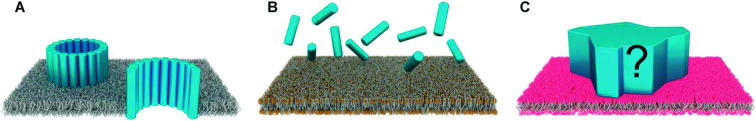
Schematic illustrations of perforin (blue) interacting with lipid substrates of different packing and charge. (A) On zwitterionic (net neutral) and disordered membranes, perforin oligomerizes into membrane spanning pores. (B) On more ordered membranes, such as liquid-ordered membranes that contain cholesterol (yellow), perforin cannot bind. (C) On membranes containing lipids with negatively charged headgroups (pink), perforin is sequestered into plaques. Membrane lesions are not formed, and the protein orientation on the surface is not known (symbolized by the question mark).

As discussed in this paper, the physical properties of membranes play essential roles in determining their sensitivity to perforin pore formation. This applies to the lipid order and packing, which reduce perforin binding to the membrane,^11,14^ and to the lipid charge, which causes perforin to be trapped in dysfunctional aggregates,^14^ as illustrated in Fig. 12. We briefly discussed membrane tension as a possible factor, which has been reported to enhance perforin function in the immune synapse.^61^

Compared with previous results, we have here (i) demonstrated the power of AFM and model membranes in investigating the lipid specificity of pore forming proteins and of perforin in particular; (ii) used AFM to demonstrate how membrane order in gel-phase lipids completely prevents perforin binding, as previously observed for liquid-ordered domains;^14^ (iii) demonstrated that this lipid specificity for liquid-disordered membranes is robust against variations in divalent ion concentration (Mg^2+^, and Ca^2+^ above the threshold needed to facilitate perforin binding to the membrane); (iv) verified the extraordinary height (*ca.* 15 nm above the membrane) of dysfunctional perforin aggregates observed on negatively charged membranes; (v) confirmed the electrostatic nature of how such membranes disable perforin; and (vi) showed that perforin pore formation is relatively insensitive to interfacial membrane tension, although it may play a role in restoring perforin functionality on PS-rich membranes.

To assess the physiological relevance of these findings, they need to be compared with cell-based assays, *e.g.*, possible correlations of perforin lysis with lipid order in target cell membranes,^12^ which confirm that reduction of lipid order sensitizes CTL membranes to perforin and that non-lytic perforin is co-localized with externalized (non-apoptotic) PS on CTLs.^14^

Finally, it is noted that related pore forming proteins have been reported to show specificity for particular lipids, *e.g.*, the membrane attack complex^30^ and gasdermin^31^ show specificity for negatively charged lipids, whereas bacterial CDCs prefer cholesterol-rich and hence liquid-ordered domains in phase separated membranes.^46^ These observations indicate a wide range of biomedically relevant processes in which the physical properties of membranes may be determinants of the function of pore forming proteins.

## Conflicts of interest

There are no conflicts to declare.

## Supplementary Material
